# Fructose Might Contribute to the Hypoglycemic Effect of Honey

**DOI:** 10.3390/molecules17021900

**Published:** 2012-02-15

**Authors:** Omotayo O. Erejuwa, Siti A. Sulaiman, Mohd S. Ab Wahab

**Affiliations:** Department of Pharmacology, School of Medical Sciences, Universiti Sains Malaysia, 16150 Kubang Kerian, Kelantan, Malaysia; Email: sbsamrah@kb.usm.my (S.A.S.); msuhaimi@kb.usm.my (M.S.A.W.)

**Keywords:** fructose, honey, hypoglycemic effect, diabetes mellitus, pancreas, liver

## Abstract

Honey is a natural substance with many medicinal properties, including antibacterial, hepatoprotective, hypoglycemic, antioxidant and antihypertensive effects. It reduces hyperglycemia in diabetic rats and humans. However, the mechanism(s) of its hypoglycemic effect remain(s) unknown. Honey comprises many constituents, making it difficult to ascertain which component(s) contribute(s) to its hypoglycemic effect. Nevertheless, available evidence indicates that honey consists of predominantly fructose and glucose. The objective of this review is to summarize findings which indicate that fructose exerts a hypoglycemic effect. The data show that glucose and fructose exert a synergistic effect in the gastrointestinal tract and pancreas. This synergistic effect might enhance intestinal fructose absorption and/or stimulate insulin secretion. The results indicate that fructose enhances hepatic glucose uptake and glycogen synthesis and storage via activation of hepatic glucokinase and glycogen synthase, respectively. The data also demonstrate the beneficial effects of fructose on glycemic control, glucose- and appetite-regulating hormones, body weight, food intake, oxidation of carbohydrate and energy expenditure. In view of the similarities of these effects of fructose with those of honey, the evidence may support the role of fructose in honey in mediating the hypoglycemic effect of honey.

## 1. Introduction

Honey is a natural substance with various medicinal properties which include antibacterial [1], antihypertensive [2], hepatoprotective [3], hypoglycemic and antioxidant effects [4]. It comprises mainly fructose and glucose along with other bioactive constituents such as assorted phenolic compounds, flavonoids, organic acids, enzymes and vitamins [5]. The fructose in honey is found to vary from 21.0% to 43.5%, while the ratio of fructose to glucose ranges from 0.46 to 1.62 [6,7,8,9,10]. These variations are due primarily to differences in floral sources, geographical origin and climatic factors [5]. Fructose is the sweetest of all naturally-occurring and available sweeteners or sugars [11]. It has a glycemic index of about 19 compared to that of glucose which is 100 [11]. Sucrose and honey have comparable glycemic indices, 61 and 58, respectively [11]. Other sources of fructose include sugar cane, sugar beets, fruits (such as dates, apples and grapes) and some vegetables (such as carrots, corns, onions and sweet potatoes) [11,12,13,14,15]. Honey supplementation has been found to reduce hyperglycemia in rodents and humans with diabetes mellitus [4,8,9,16]. However, the mechanisms of the hypoglycemic effect of honey remain unclear. The possible roles of fructose, mineral ions (such as zinc, copper and vanadium), phenolic acids and flavonoids have been suggested [4,8,9,16,17]. The protection of the pancreas against oxidative stress and damage (via honey antioxidant molecules such as organic acids and phenolic compounds) is one such potential mechanism [18].

The objective of this review is to summarize findings on the hypoglycemic effect of fructose. The data indicate that fructose enhances hepatic glucose uptake via activation of glucokinase and promotes synthesis and storage of glycogen via activation of glycogen synthase in the liver. The findings indicate that glucose and fructose might exert a synergistic effect in the intestine and pancreas. This might enhance intestinal fructose absorption in the intestine and stimulate insulin secretion in the pancreas. The studies reveal that fructose might improve glycemic control independent of its insulinotropic effect. The data demonstrate the beneficial effects of fructose on glucose- and appetite-regulating hormones, glycemic response, body weight, food intake, oxidation of carbohydrate and energy expenditure. On the basis of the similarities of these effects of fructose with those of honey, even though data regarding the effects of honey are still limited, the evidence may support the role of fructose in honey in contributing to the hypoglycemic effect of honey. Thus, the possibility that fructose in honey might mediate the hypoglycemic effect of honey merits scientific investigation.

## 2. Overview of Fructose (and in Relation to Honey) in the Gastrointestinal Tract (GIT)

The GIT is an important barrier that plays a vital role in determining the biological or pharmacological effects of many orally administered agents by influencing their absorption and bioavailability [19]. Generally, carbohydrates are hydrolyzed by the intestinal brush border hydrolases to generate monosaccharides (glucose, fructose and galactose) before they are absorbed [19]. Glucose and galactose are taken up via the SGLT1, a Na^+^/glucose (galactose) co-transporter [19]. In contrast, fructose is transported across the apical membrane by GLUT5 and/or GLUT2 via facilitated diffusion, though some evidence suggests uptake may be via active transport [19,20]. Unlike glucose and galactose, fructose delays gastric emptying, which may inhibit food intake, leading to its slower absorption [19,21,22]. Glucose and/or fructose can upregulate GLUT2 mRNA expression [21]. In contrast, GLUT5 mRNA transcription is upregulated by fructose only and thereby enhances fructose absorption [23]. Studies have shown that glucose and/or galactose can enhance fructose absorption [12,14,15]. While the mechanisms are not yet fully understood, it is suggested that in the presence of glucose, there is combined absorption of the two monosaccharides, reminiscent of a disaccharidase-related transport system [24]. Some findings suggest that glucose facilitates fructose absorption via passive diffusion [25], whereas others support the recruitment of GLUT2 to the brush border membrane in response to increased intestinal fructose [25].

In summary, evidence indicates that fructose has a special carrier different from that of glucose [19,20]. Studies show that the presence of fructose increases this transporter resulting in increased fructose absorption [23]. The presence of glucose further enhances fructose absorption [12,14,15,24,25]. All these findings could be very important in regard to honey. This is because honey consists of primarily fructose and glucose. Hence, there is a possibility that administration of honey might increase the transcription of fructose transporter thereby enhances fructose absorption [23]. The glucose in honey might also facilitate fructose absorption [12,14,15,24,25]. Moreover, recent data indicate that gut microbiota enhances the intestinal absorption of monosaccharides including fructose [26]. Interestingly, honey comprises oligosaccharides which enhance the activity and growth of gut microorganisms [27]. Therefore, it is possible that enhanced activity and growth of gut microbiota due to honey supplementation might also contribute to increased intestinal absorption of honey fructose.

## 3. Overview of Fructose (and other Monosaccharides) in the Liver

After absorption, monosaccharides are transported to the liver which plays a key role in glucose homeostasis [28]. In the liver, the uptake and initial steps of metabolism of glucose and fructose differ [22]. For instance, insulin is required for the hepatic uptake of glucose, but not for fructose [22]. It is known that larger amounts of fructose than glucose are extensively metabolized in the liver [19]. This differential metabolism might result in more glucose than fructose passing through the liver with reduced metabolism [19]. Previous reviews have described in details the metabolism of fructose and other monosaccharides [22,29]. Therefore, what is presented here is a summary to provide the necessary knowledge to understand how metabolism of these monosaccharides (glucose and fructose) might contribute to hypoglycemia. Glucose is phosphorylated by glucokinase in the liver to obtain glucose 6-phosphate [29]. This is the first rate-determining step in the metabolism of glucose. Metabolism of glucose 6-phosphate by phosphofructokinase produces fructose 6-phosphate, the second rate-limiting step [29]. Fructose 6-phosphate is converted to fructose 1,6-diphosphate by phosphofructokinase, which is further metabolized by aldolase to dihydroxyacetone and glyceraldehyde 3-phosphate. In each of these different catalytic reactions, insulin plays an important role [29].

In contrast, galactose is converted to galactose 1-phosphate by galactokinase. Metabolism of galactose 1-phosphate, which is catalyzed by phosphoglucomutase, produces glucose 1-phosphate which then enters the glycolytic pathway [29]. However, in the case of fructose which is extensively metabolized into fructose 1-phosphate, the reaction is catalyzed by fructokinase [22,29]. The high hepatic extraction of fructose results in excessive production of fructose 1-phosphate which inhibits glycogenolysis [22,29]. This enhances the conversion of fructose into lactate [22,29]. The enzyme aldolase then converts fructose 1-phosphate to dihydroxyacetone phosphate and glyceraldehyde, which are glycolytic substrates [22,29]. Through the activity of aldolase, condensation of dihydroxyacetone phosphate with glyceraldehyde 3-phosphate may produce fructose 1,6-diphosphate, yielding glucose or glycogen [22,29]. Dihydroxyacetone phosphate may also be reduced to glycerol-3-phosphate, a substrate for triacylglycerols and phospholipids [22,29]. Unlike in glucose metabolism, all these catalytic reactions occur independently of insulin and the rate-limiting steps are also bypassed in fructose metabolism [22,29]. These differences in metabolism result in about 50% to 70% of the absorbed fructose being metabolized in the liver [29], compared to only about 20% to 30% of the absorbed glucose [30]. A simplified figure that summarizes these pathways of fructose metabolism in the liver has already been presented by Waford [31]. Interested readers are referred to see reference no 33 for further details [31].

In summary, as highlighted in this section, this differential metabolism of fructose and glucose in the liver seems very relevant. This is in view of the fact that honey is enriched in both fructose and glucose [5,6,7,8,9,10]. As will be explained later, the liver is the major site where fructose exerts its hypoglycemic effect [31,32,33]. Compelling evidence indicates that both glucose and fructose act synergistically in the liver to elicit hypoglycemic effect [33,34,35]. Considering that more of the absorbed fructose is phosphorylated in the liver than the absorbed glucose [29,30], similar proportion of fructose and glucose in honey might also be phosphorylated in the liver. Should that be case, with the activation of glucokinase and other enzymes involved in glycogenesis by fructose, more of the previously unmetabolized glucose might be taken up again from the circulation into the liver. With larger quantities of fructose undergoing continuous and extensive metabolism in the liver than glucose [29,30], this might contribute to further or additional uptake of glucose from the circulation. In other words, honey supplementation (via its fructose) might enhance glucose uptake, synthesis and storage of glycogen in the liver of diabetic rodents or humans. This would result in improved glycemic control in diabetes mellitus. Studies have also shown that honey administration ameliorates hepatic oxidative stress and produces hepatoprotective effect [16,36,37]. These antioxidant and hepatoprotective effects might be beneficial to the liver, especially in diabetes mellitus. These effects might improve liver efficiency in metabolizing honey fructose and thereby contribute to hypoglycemic effect of honey via improved hepatic enzymes involved in glucose metabolism.

## 4. Effects of Fructose in the Liver

The liver plays an important role in glucose regulation [22,28]. As explained earlier, it also has a potential to mediate the glucose-lowering effect of honey fructose [31,32,33]. A number of studies have investigated the effects of fructose, either alone or together with glucose, in rodents or their excised livers. In isolated hepatocytes, addition of a small amount of fructose activates glucokinase and increases the rate of glucose phosphorylation [38,39]. The role of hepatic glucokinase in mediating the hypoglycemic effect of fructose is also corroborated by Nishi *et al.* [38]. The authors reported that low doses of fructose produced no effect on phosphorylation of glucose or glycolytic flux in the diabetic hepatocytes that lacked glucokinase [38]. A similar lack of effect was also reported in the diabetic hepatocytes which expressed glucokinase, but was incubated with a glucokinase inhibitor (mannoheptulose) [38]. Similarly, glucose and fructose added to isolated perfusion of liver produced synergism [32,34].

Administration of fructose was reported to increase hepatic glucose and fructose uptake, glucose 6-phosphate, fructose 1-phosphate, glycogen synthesis, glycogen deposition and hepatic lactate production in the liver of rodents or dogs [31,35,40,41]. These hepatic effects of fructose may result in reduced postprandial hyperglycemia and/or suppressed insulin secretion by the pancreatic beta-cells [35,41]. The role of fructokinase is also implicated in the glucose-lowering effect of fructose [31,38,39]. The glucose-lowering effect of fructose is also attributed to increased expression or activation of some enzymes such as glucose6-phosphate dehydrogenase, aldolase B, phosphofructokinase-1 and glycogen synthase and inhibition of glucose 6-phosphatase and phosphorylase [32,33,34,38,39,42]. This results in increased hepatic glycogen synthesis and storage [32,33,34,38,39,42]. By and large, these findings indicate that small amounts or catalytic doses of fructose are capable of markedly increasing hepatic glucose uptake and glycogen synthesis and deposition via activation of glucokinase and other enzymes or inhibition of some enzymes [31,32,33,34,38,39,42]. These hepatic effects of fructose lead to improved glucose tolerance and reduced elevated blood glucose [35,41]. It is worth mentioning that the beneficial effects of fructose on hepatic glycolytic enzyme phosphorylase are observed only with small or moderate doses (2.22 µmol/kg/min) [31].

## 5. Effects of Fructose in the Pancreas

The pancreas, which secretes two key glucose-regulating hormones—insulin and glucagon—is an important organ in diabetes mellitus [43]. Many drugs and natural products such as plant extracts exert their hypoglycemic effect by acting on pancreas. Fructose is not an exception either. Evidence suggests that any sugar capable of stimulating insulin secretion from the pancreas must first be metabolized in the islet cells [44,45]. Studies have shown that both fructose and glucose are capable of stimulating insulin secretion in perfused rat pancreas preparations [44,45]. In contrast, other sugars such as galactose, xylose and L-arabinose do not stimulate insulin release from isolated rat pancreas preparations [44,45]. However, reports suggest that glucose is a better substrate than fructose [44,45]. The ability of fructose to stimulate insulin release from isolated rat pancreas preparations depends on glucose concentrations [46,47]. However, some studies reported that fructose did not stimulate insulin secretion in isolated rabbit or rat pancreatic islets [48]. Taken together, these studies indicate that the amount of insulin release is dependent on the extent to which sugars can be metabolized in pancreatic islets. The findings also suggest while fructose may stimulate insulin release from pancreas, its ability to stimulate insulin secretion is limited.

## 6. Effects of Fructose on Glycemic Control and Glucose-Regulating Hormones

Glucose, unlike fructose, is a major physiological regulator of biosynthesis and secretion of insulin [43]. A number of studies have investigated the effects of fructose on parameters relating to glycemic control and glucose-regulating hormones. In normal rats, fructose administered alone or as sucrose was reported to improve glucose homeostasis and insulin response compared with rats administered glucose alone [49]. Similarly, studies have shown that fructose supplementation in normal rats or type 2 model of diabetic rats produced lower levels of plasma insulin and glucose more than did other sugars [50,51]. In dogs, inclusion of small amounts of fructose with a glucose load was shown to reduce insulin secretion from the pancreatic beta-cells [35].

In human subjects, data on the effect of fructose on glycemic control and glucose-regulating hormones are inconsistent. A number of studies demonstrated that fructose ingestion (7.5 g) or fructose-enriched meals (25% of energy requirements as fructose) markedly reduced plasma glucose, serum fructosamine, serum glycated hemoglobin, serum glycosylated albumin and serum insulin in healthy, impaired glucose-tolerant, overweight, obese, type 1 and type 2 diabetic subjects [52,53,54,55,56,57]. Low or moderate doses (0.25, 0.5, 0.75 or 1.0 g or 3.5 µmol/kg/min) of fructose intake or infusion also increased glycogen synthesis, glycogen synthase flux and endogenous lactate and pyruvate production [58,59,60]. Besides, it was reported that consumption of fructose-sweetened beverages with meals lowered the levels of insulin and blood glucose in normal-weight, obese men and women [61]. Some complex carbohydrates, which are rich in fructose [11,12,13,14,15], are known to markedly lower the elevations in blood glucose and plasma insulin compared to simple sugars in type 2 diabetic patients [62]. However, some studies found no effects of moderate or even high doses (3.5 g fructose/kg fat-free mass/day) of fructose ingestion or infusion on serum/plasma levels of glucose, postprandial plasma glucose, glycated hemoglobin, glycosylated albumin, insulin and insulin sensitivity in healthy, lean, obese non-diabetic, obese or type 2 diabetic subjects [63,64,65]. Findings suggest that the ability of fructose to stimulate insulin secretion may depend on the level of circulating glucose [66,67]. Nevertheless, it is also worth mentioning that some studies have associated fructose consumption or feeding with elevated glucose, impaired glucose tolerance, elevated insulin concentrations, decreased insulin sensitivity and insulin resistance [68,69]. However, these effects were observed only with increased or high fructose consumption or feeding (3.5 g fructose/kg fat-free mass/day) [68,69].

## 7. Effects of Fructose on Appetite-Regulating Hormones

The role of fructose is implicated in the modulation of appetite-regulating hormones such as ghrelin and leptin. Ghrelin is a 28 amino acid peptide hormone produced in the stomach that stimulates hunger [70]. Its levels increase before meals and decrease after meals [70]. Similarly, leptin is a 167 amino-residue peptide hormone secreted by adipose tissue [70]. It plays an important role in the regulation of appetite, food intake and energy expenditure [70]. Its secretion is influenced by circulating levels of insulin [70]. A study by Teff *et al.* [61] reported that fructose ingestion (30% of energy requirements as fructose) reduced the levels of leptin in normal-weight women while no such effect was observed with glucose consumption [61]. The change in the concentrations of leptin between the morning nadir and the late night peak was also reduced following fructose consumption [61]. Another study in obese subjects found that consumption of fructose-sweetened beverages was associated with reduced circulating levels of leptin [71]. Lowered concentrations of circulating insulin and/or glucose resulting from fructose consumption may cause reduced serum leptin, which may contribute to weight gain [61,72]. However, a study reported that fructose (compared to glucose) did not reduce or increase leptin level [73], while high fructose consumption (1.5 g fructose/kg body weight) was found to increase fasting levels of leptin [74]. Besides the possibility of fructose consumption causing reduced levels of leptin, leptin resistance has been reported with the consumption of high fructose diet in rats [75,76]. Leptin resistance is a phenomenon whereby elevated levels of leptin failed to reduce appetite or mediate weight loss [75,76]. In a nutshell, these studies indicate that low or moderate doses (30% of fructose-derived kilocalories) of fructose reduce leptin levels [61,71], whereas increased or high consumption (1.5 g fructose/kg body weight or 60% fructose diet) of fructose increases leptin levels [74,75,76].

## 8. Effects of Fructose on Body Weight, Food Intake, Oxidation of Carbohydrate and Energy Expenditure

Similar to other parameters, fructose consumption or feeding also influences body weight and food/energy intake. In rats, high fructose feeding resulted in increased weight gain [77,78]. A similar finding was also reported in mice [79]. An evidence-based review of literature revealed that normal or moderate dietary consumption of fructose does not cause weight gain in overweight and obese individuals [80]. Findings from another recent study showed that a low- (<20 g/day) or moderate (50–70 g/day)-fructose diet with natural fruit supplements in obese subjects caused weight loss compared with baseline [81]. The study also indicated that the moderate-fructose diet with natural fruit supplements markedly reduced weight loss more than did the low-fructose diet [81]. However, some studies have linked increased consumption of fructose- or sugar-sweetened beverages to excess calorie intake and increased body weight [82,83]. On the other hand, some studies found no significant effect of fructose on body weight [76,84,85]. Findings indicate that fructose suppresses food or energy intake in rats [86,87]. Similarly, a study that compared the effect of preloads of 50 g of glucose or fructose showed that fructose-preloaded subjects consumed fewer calories and less fat than did glucose-preloaded subjects [88]. Similar results were also reported in healthy, lean, obese and type 2 diabetic subjects [89,90].

The effects of fructose on oxidation of carbohydrate and energy expenditure have also been investigated. A study showed that in healthy volunteers, fructose elicited a greater increase in oxidation of carbohydrate and energy expenditure than did glucose [91]. Similar results were also reported in young control subjects [92]. Schwarz *et al.* showed that diet-induced thermogenesis and oxidation of carbohydrate were considerably greater with fructose than with glucose [93]. It is suggested that increased energy expenditure following fructose consumption may be due to increased carbohydrate oxidation; and the fact that conversion of fructose to glycogen requires more energy than that of glucose to glycogen [22]. Similarly, in healthy lean male volunteers, fructose and sucrose elicited greater increments in carbohydrate oxidation and total energy expenditure than did glucose and starch [94]. Similar findings were reported during exercise [95]. Hence, these studies indicate that fructose may increase or reduce body weight depending on the doses. The findings also reveal that fructose feeding suppresses food or energy intake and increases carbohydrate oxidation and energy expenditure. Thus, these data suggest that if fructose is taken at moderate doses (<20 g/day or 50–70 g/day), it has a potential to reduce but not increase weight gain.

## 9. Effects of Honey which are Similar to Those of Fructose

By and large, these findings on the effects of fructose are very remarkable. This is in view of the fact that honey comprises predominantly fructose and glucose [5,6,7,8,9,10]. A study by Münstedt *et al*. showed that honey intake (75 g) increased serum levels of fructose in healthy humans [10]. However, small variations in fructose-to-glucose ratio of honey varieties may not make much difference in glycemic and/or insulinemic indices [6,10,96]. A study that compared the effects of honey and a honey-comparable glucose-fructose solution found that honey supplementation significantly lowered serum concentrations of glucose, insulin and C-peptide than the honey-comparable glucose-fructose solution in healthy subjects [97]. A study by Deibert *et al.* also supports the potential role of fructose in mediating the hypoglycemic effect of honey [7]. In their study, the authors found that the fructose content of honey, rather than its fructose-glucose ratio, was negatively correlated with the glycemic index [7]. Similarly, subjects with normal glucose tolerance, impaired glucose tolerance, mild diabetes or type 2 diabetes mellitus were reported to exhibit markedly lower serum/plasma concentrations of glucose, insulin and C-peptide after honey supplementation than after dextrose, sucrose or simulated honey [98,99,100]. These data are similar to those reported for fructose in subjects with normal or impaired glucose tolerance or diabetes in whom fructose significantly reduced serum/plasma levels of glucose, insulin and C-peptide [52,61,71]. Similar to findings obtained with fructose [63,64], some studies also found no significant effect of honey on serum/plasma levels of glucose and insulin in diabetic patients [101,102].

A study found that, in both alloxan- and fructose-induced diabetic rats, honey feeding (10 mL honey/kg/5 mL distilled water) for three weeks resulted in reduced blood glucose concentrations [103]. Also, administration of honey (1.0 g/kg body weight) was reported to reduce serum levels of glucose and fructosamine in diabetic rats [9]. Considerable improvement in pancreatic islets and increased serum insulin levels were reported in honey (1.0 g/kg)-treated diabetic rats [9,51,104]. In non-diabetic rats, reduced glycated hemoglobin was reported after honey (10%) supplementation [105]. However, a study did not find any significant difference in concentrations of glucose and insulin in normal rats fed honey-based diet and sucrose [106]. This may be due to the similar proportion of fructose in both honey and sucrose. These findings also corroborate ours in which we found that honey supplementation (1.0 g/kg body weight) in non-diabetic rats produced no significant effects on the levels of serum insulin, glucose and fructosamine [9]. Similarly, pancreatic islets of normal rats treated with honey did not differ from those of untreated normal rats [104]. 

Studies have also shown that honey supplementation (10 or 20%) significantly reduced body weight gain and food/energy intake in rats [105,106,107]. In humans, honey was found to mildly decrease body weight while it does not increase body weight in overweight or obese subjects [108]. These data are comparable to the effects reported for fructose in rats [86,87] and overweight or obese subjects [80,81]. A recent study showed that the levels of leptin in rats administered honey were considerably lower than in those given sucrose [106]. Similar observations or findings were also documented for fructose [61,71]. Larson-Meyer and colleagues showed that honey, compared with sucrose-containing meal, delayed postprandial ghrelin response and enhanced the total peptide YY response [109]. Peptide YY is a protein secreted by cells in the ileum and colon in response to food ingestion or intake, and suppresses appetite [109]. Taken together, the similarities of findings of the effects of fructose and honey suggest that hypoglycemic effect of honey might depend partly on the fructose content of honey.

## 10. Conclusions and Future Perspectives

These studies indicate that the presence of fructose increases its transporter levels resulting in increased fructose absorption. Besides, evidence reveals that the presence of glucose enhances fructose absorption. The review also presents findings that support a possible synergistic effect of glucose on fructose in stimulating insulin release from the pancreas. It also presents data that demonstrate the beneficial effects of fructose in the liver. Even though the data or findings on the effects of fructose show some discrepancies, the majority of the data indicate that low or moderate doses of fructose exert beneficial hepatic effects such as activation of hepatic glucokinase, enhanced hepatic glucose uptake, increased hepatic glucose6-phosphate, activation of hepatic glycogen synthase, increased glycogen synthesis and deposition. These hepatic effects would suffice to elicit improved glycemic control. The consistency of data on the effects of fructose in the liver, despite little or no insulinotropic effect, suggests that fructose acting through the liver might play a role in the hypoglycemic effect of honey. Therefore, based on the similarities of findings of the effects of fructose and honey, and coupled with the fact that honey comprises mainly fructose and glucose, the evidence may support the role of fructose in mediating the hypoglycemic effect of honey. Therefore, studies that investigate the potential role of fructose in the euglycemic and hypoglycemic effects of honey are warranted. Besides, further studies that unravel the potential role of liver in mediating the hypoglycemic effect of honey are recommended. With this review, we have not excluded the prospect of a yet to be identified substance in honey contributing to improved glycemic control. In view of limited data, we recommend randomized, controlled studies in diabetic and non-diabetic human subjects to determine the effects of honey (and its graded doses) on glycemic control (glucose and fructosamine/glycated hemoglobin), glucose-regulating hormones (insulin and glucagon), appetite-regulating hormones (leptin and ghrelin), weight gain, calorie intake and energy expenditure. 
